# Evaluating the effectiveness of opportunistic eye screening model for people with Diabetes attending Diabetes clinic at Mnazi Mmoja hospital, Zanzibar

**DOI:** 10.1186/1471-2415-14-81

**Published:** 2014-06-24

**Authors:** Fatma J Omar, Sethu Sheeladevi, Padmaja Kumari Rani, Geng Ning, George Kabona

**Affiliations:** 1 Gullapalli Pratibha Rao International Center for Advancement of Rural Eye Care, L V Prasad Eye Institute, Hyderabad, India; 2 Mnazi Mmoja Referral Hospital, Zanzibar, Tanzania; 3 School of Optometry & Vision Science, University of New South Wales, New South Wales, Australia; 4 Srimati Kannuri Santhamma Centre for VitreoRetinal Diseases, Kallam Anji Reddy Campus,L V Prasad Eye Institute, L V Prasad Marg, Hyderabad, India; 5 Iringa Regional Referral Hospital, Iringa, Tanzania

**Keywords:** Diabetic retinopathy, Screening model, Zanzibar

## Abstract

**Background:**

Diabetes and its related microvascular complications like Diabetic retinopathy are showing an alarming rise in developing countries like Zanzibar. Objective of the present study is to evaluate the impact of integrating eye screening for all subjects attending the diabetes clinic at Mnazi Mmoja Hospital in Zanzibar and to estimate the prevalence of visual impairment and diabetic retinopathy among the subjects.

**Methods:**

This is a cross sectional study involving 356 randomly selected patients who had attended the diabetes clinic between July and August 2012. All subjects underwent comprehensive eye examination including fundus evaluation after dilatation by a cataract surgeon and an ophthalmologist, independently. Data was collected using the designated questionnaire and analysed using the SPSS software. Blindness and visual impairment was defined as presenting VA <3/60 and <6/18 to 6/60 in the better eye respectively and DR was graded using the International classification of Diabetic Retinopathy severity grading scale.

**Results:**

A total of 356/967 subjects were recruited in a duration of 2 months; 176 (49.4%) were male and the mean age was 52.21 (SD 15.3). Targeted eye screening of diabetics showed that 231/356 (65%) of the subjects had eye problems, including potentially blinding conditions that required immediate intervention in contrast to the existing self reported referral where only 10% of an average of 200 diabetics underwent eye checkup in a year. The prevalence of visual impairment was 20.2%; 95% CI: 16.4-24.7 and blindness in 9.3%; 95% CI: 6.7 -12.7. The prevalence of DR was 28.3% and sight-threatening DR was reported in 9%. Among the DR cases, 30% had sight threatening DR including 28% macular edema, 2% severe NPDR and PDR. Multivariate analysis showed a higher risk for any DR in older subjects >50 years (OR: 2.19; 95% CI: 1.14 – 4.25) and in females (OR: 1.92; 95% CI: 1.07 – 3.44).

**Conclusion:**

Opportunistic DR screening model achieved higher yield of identification of visual impairment and DR compared to the yield of 10% of existing self reported Diabetic eye screening model at Zanzibar. Integration of eye screening at diabetes clinics helps in early identification and provision of appropriate treatment for reducing blindness due to diabetes.

## Background

Diabetes is a major public health problem in both the developed and the developing countries, particularly type 2 diabetes, which is rising in epidemic proportions [1]. Diabetic retinopathy is the fifth leading cause of global blindness and an important cause of preventable blindness if detected at an early stage [2]. Currently, Africa is estimated to have more than 7 million people with Diabetes and these figures are expected to be nearly 12.2 million by 2030 [3,4]. More than 482,000 East Africans are now diagnosed to have type 2 diabetes [5].

Zanzibar is a part of the United Republic of Tanzania. It has 1.2 million inhabitants according to the 2002 Country census survey [6]. Diabetes, and its related complications like diabetic retinopathy are increasing rapidly in Zanzibar and other African countries [7] due to inadequate knowledge about the disease and its associate complications among the communities and health workers. Moreover, there have been substantial changes in lifestyles and an increase in urbanization and in reduction of physical activities [8].

Unfortunately, due to the asymptomatic nature of diabetic retinopathy in the early stages, many patients present late, when the damage is already rendered irreversible. In the African region, a few hospital based DR prevalence studies suggested prevalence estimates, ranging from 7% to 63% [9,10]. It is estimated that more than 50% of DR cases are undiagnosed in the community.

There is no previously published data on DR screening models in Zanzibar. This calls for urgent initiatives to promote education about diabetes and its related complications, advocate for diabetes screening campaigns and develop an effective DR screening model suitable for Zanzibar.

WHO recommends dilated regular eye examination for all persons with diabetes to identify eye problems at an early stage and intervene to avoid blindness. An important feature of an effective DR screening model is to have DR screening for the people with Diabetes at their first contact that is a physician (i.e. Opportunistic DR screening). The current referral pattern to the eye clinic at Zanzibar is based on the self-reported eye complaints by the diabetics. Currently, there are no facilities for diagnosis or medical and surgical management of DR at the eye unit in Zanzibar.

The present study is meant to find the efficacy of an opportunistic DR screening model in terms of the yield of DR, sight threatening DR and other causes of visual impairment among people with Diabetes attending a Government Diabetes clinic in Zanzibar.

## Methods

This was a hospital-based cross sectional study involving 356 randomly selected patients who had attended a Mnazi Mmoja Hospital diabetes clinic from July to August 2012. Mnazi Mmoja Referral Hospital is the only tertiary hospital in the country with facilities of various departments that treat different cases.

The subjects were randomly recruited at the exclusive diabetic outpatient’s clinic at the Mnazi Mmoja Referral Hospital. The investigator approached all the patients attending the diabetic clinic during the study period daily and explained about the study. All those consented to participate in the study was further interviewed before the eye examination. All those diabetic patients who were not interested to participate and who was seriously ill and had difficulty to move to the eye clinic for examination was excluded from participation.

Informed consent was obtained from all participants prior to recruitment in the study. Ethical approval was obtained from Zanzibar Medical Research Ethical Committee.

The study participants were interviewed and the information was recorded using a designated questionnaire by the investigator. Systemic parameters were measured by trained nurses at the Diabetes clinic included blood sugar (mmol/l) by digital Glucoplus machine, blood pressure by digital BP machine (mmHg), weight (in Kgs), height (in meters) and waist circumference (in meters). Fasting blood sugar was classified as within normal limits ≤ 6 mmol/l) and high as > 6 mmol/l and blood pressure was classified as presence of hypertension ≥ 140/90 mm of Hg and absence of hypertension when blood pressure is within normal limits (120/80 – 110/75 mmHg). BMI was considered as within Normal (18.50- 24.99), overweight (25–30) and Obese if the value was more than 30.

Ophthalmic Clinical Officers (Ophthalmic assistants) were responsible for testing visual acuity with Snellen vision chart at a distance of 6 metres. Presenting visual acuity of 6/18- 6/6 was considered as a normal vision, presenting vision of <6/18 in the better eye was defined as visual impairment, and presenting vision of <3/60 in the better eye as blindness. Anterior segment examination by slit lamp and Intra ocular pressure using digital tonopaf machine was done for all the subjects, prior to pupillary dilatation by the cataract surgeons (cataract surgeon is a non-ophthalmologist mid-level eye care professional who is trained in general ophthalmology and takes a course in cataract surgery for 1 year at a recognized institution and he/she only performs simple cataract surgeries). Pupils were dilated using 1% tropicamide eye drops. Fundoscopy examination was performed independently by a cataract surgeon and an ophthalmologist using indirect ophthalmoscope and 20Ds lens after full dilatation of the subject’s pupil. Over 80% agreement was achieved between the two ophthalmic clinical officers for visual acuity assessment, the two cataract surgeons for anterior segment examination before the start of main study. There was perfect agreement between the cataract surgeon and the ophthalmologist in categorizing patients with different types of diabetic retinopathy (DR), Kappa = 0.993 (95% Confidence Interval (CI): 0.980, 1.000).

Diabetic retinopathy (DR) was graded using the International classification of Diabetic Retinopathy severity grading scale [11] as No DR, Mild non proliferative DR (mild NPDR), Moderate non proliferative DR (moderate NPDR), Severe Non Proliferative DR (NPDR), Proliferative diabetic retinopathy (PDR) and Macular edema. Sight threatening retinopathy was defined as any eye having Severe NPDR, PDR and Macular edema.

All data collected was entered into a database and analysed using the SPSS software 16.0 version. The prevalence of visual impairment, eye diseases and diabetic retinopathy was calculated for different age groups and gender. Univariate and multivariate regression analysis was used to determine the factors associated with DR and a p value <0.05 was considered statistically significant. The data obtained during the study was correlated with the past statistics maintained at the hospital to report the effectiveness of this methodology.

## Results

A total of 356 subjects were recruited out of 967 patients who had attended the diabetes clinic during the study period. The mean age of subjects was 54.27 years (SD 12.7) and 50.6% (180) were women and 5% were children. The socio economic characteristics of the study subjects gender wise is presented in Table 1.

**Table 1:** Socio-demographic characteristics of study subjects by gender

**Socio-demographic status**	**Female (%)**	**Male (%)**	**Total (%)**	**P value**
**Age group**				
6 –18 yrs	11 (3.1)	7 (2.0)	18 (5.1)	**0.18**
19 – 49 yrs	53 (14.9)	58 (16.3)	111 (31.2)	
50 and above	112 (31.5)	115 (32.3	227 (63.6)	
**Educational level**				
Never been to school	81 (23)	33 (9.3)	114 (32.3)	**<0.001**
Primary & secondary school	98 (27.5)	131 (36.8)	229 (64.4)	
Graduate and above	2 (0.6)	11 (3.2)	13 (3.8)	
**Occupation**				
Govt .employees & Entrepreneurships	41 (11.5)	119 (33.5)	160 (45.0)	**<0.001**
Students	6 (1.7)	13 (3.7)	19 (5.4)	
Retired from government	5 (1.7)	32 (9)	37 (10.4)	
Housewife/non-workers	128 (36.1)	12 (3.4)	140 (39.4)	
**Location**				
Urban	78 (21.9)	80 (22.5)	158 (44.4)	**0.43**
Rural	92 (25.8)	81 (22.8)	173 (48.6)	
Semi-urban	10 (2.8)	15 (4.2)	25 (7.0)	

**Figure 1:**
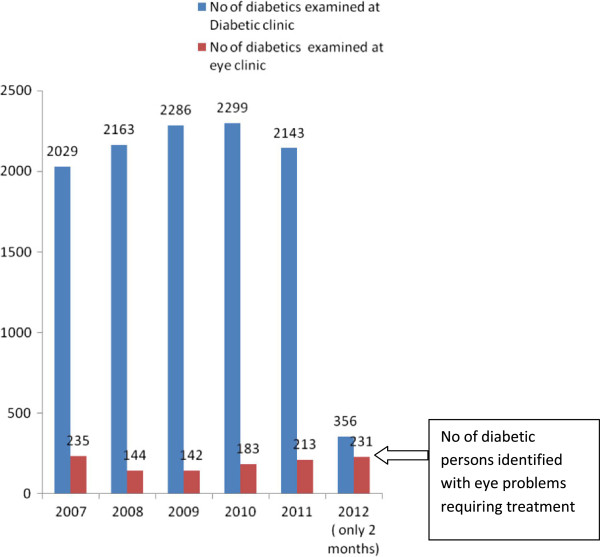
Comparative statistics of diabetic persons attending diabetes and eye clinic in the past 5 years with the study period.

A majority of the subjects had Type 2 diabetes — 315 (89%) and the mean duration of diabetes was 6.86 years (SD 5.59 years). The prevalence of known hypertension among the subjects was 199 (56%); and it was more in females as compared to males (32% vs 24%; p 0.004). The mean duration of hypertension was 7.14 years (SD 6.57 years). A total of 155 (43%) subjects had a BMI above 25, and obesity (BMI >30) was significantly higher among females as compared to males (26.1% vs 7.4%; p <0.001).Figure 1 shows the comparison of patients with diabetes attending diabetic and eye clinic in the same tertiary hospital. We compared the number of persons attending the diabetes clinic and the number of diabetic cases with eye problems that attended the eye clinic in the last five years.

During this study period of two months, 231/356 (65%) of the subjects with Diabetes having eye problems that requires further treatment were identified. In contrast, it was observed that an average of only 10% (Range 142–200 subjects) of individuals with diabetes who had reported having eye problems attended the eye clinic every year.

A total of 206/356 (57.9%) subjects reported having experienced eye problems at the time of the interview, of whom 58% were female. Of these, 61 (30%) had never accessed eye care services earlier, despite having eye problems, 71 (34%) had accessed only once and 74 (36%) had accessed more than once earlier.

Table 2 shows the prevalence of visual Impairment and blindness among the study subjects. The prevalence of Visual Impairment was seen in 20.2%; 95% CI: 16.4 -24.7 and blindness was seen in 9.3%; 95% CI: 6.7 -12.7 of subjects. About 5% of the subjects vision improved with pin hole. Prevalence of eye problems was recorded in 59% of the subjects. Cataract was diagnosed in 178 eyes and high IOP > 21 mmHg in 59 eyes.

**Table 2:** Prevalence of visual impairment and blindness among the subjects

**Categories**	**Presenting visual acuity n (%)**	**95% CI**
Normal (6/6 – 6/18)	251 (70.5)	65.6 – 75.0
Moderate VI (<6/18 – 6/60)	72 (20.2)	16.4 – 24.7
Blind (<3/60 to no perception of light)	33 (9.3)	6.7 – 12.7
Total	356 (100)	

The prevalence of DR was found in 101 (28.3%) subjects and sight-threatening DR was reported in 33 (9%) subjects with Diabetes. Among the DR cases, 56% had mild DR, 12% had moderate, 2% had severe DR or proliferative diabetic retinopathy (PDR) and 28% had macular oedema. The multiple logistic regression analysis (Table 3) shows that persons aged >50 years had a higher risk of developing any DR, as compared to <50 years age (OR: 2.19; 95% CI: 1.14 – 4.25) and the risk was statistically significant. Females were more at risk as compared to males and increase in duration of diabetes was significantly associated with any DR and sight-threatening DR. Persons who presented with visual impairment had twice the risk for any DR (OR: 2.71; 95% CI: 1.56 – 4.7) and thrice for sight-threatening DR (OR: 3.75; 95% CI: 1.61 – 8.72).

**Table 3:** Factors associated with diabetic retinopathy (DR) using multiple logistic regression

**Variables**	**Total n = 352 (%)**	**Adjusted odds ratio (95% CI)**	**P value**
**Age (years)**			
< 50 years	128 (36)	1	0.020
>50 years	224 (64)	2.19 (1.14 – 4.25)	
**Gender**			
Male	174 (49)	1	0.028
Female	178 (51)	1.92 (1.07 – 3.44)	
**Body mass index**			
Normal range	200 (57)	1	0.17
Overweight	93 (26)	1.53 (0.84 – 2.79)	
Obese	59 (17)	1.61 (0.78 – 3.32)	0.19
**Diabetes duration**			
<5 years	176 (50)	1	0.17
6 -10 years	109 (31)	1.53 (0.83 – 2.82)	
11 – 15 years	38 (11)	4.06 (1.76 – 9.36)	0.001
16 and above	29 (8)	2.67 (1.08 – 6.58)	0.03
Present history of hypertension			
No	156 (44)	1	0.33
Yes	196 (56)	1.35 (0.74 – 2.44)	
**Fasting blood sugar**			
Normal range	54 (15)	1	0.88
High	298 (85)	1.06 (0.52 – 2.14)	
Visual acuity*			
Normal range	250 (70)	1	<0.001
Visually impaired	102 (30)	2.71 (1.56 – 4.7)	

*4 persons had dense cataract and there was no view, hence they are not included for this calculation.

## Discussion

Currently, the model of self reported Diabetes eye screening at Zanzibar yields only less than 10% eye patients of the persons attending the diabetic clinic, who access the eye clinic located within the same hospital premises. The reason for the low uptake of eye care services by the diabetic population may be due to lack of awareness among the diabetic community and insufficient counseling on the importance of eye screening, as it is not a mandatory part of the treatment protocol.

Our study of opportunistic eye screening at the Diabetes clinic showed a significant yield of people with diabetes (65%) having eye problems as compared to only 10% of current self reported eye screening for people with Diabetes at Zanzibar. This study found that the prevalence of DR was 28.3% (101/356) and sight-threatening diabetic retinopathy was 9% (32/356) among the study subjects. Our findings match with previous reports from Africa on DR prevalence ranging from 16 – 55% [12].

We found perfect agreement between the cataract surgeon and the ophthalmologist, as the ophthalmologist was the only Gold standard, and it indicates that if there is continuous training and upgrading of these cadres, going forward, cataract surgeons can help detect DR in the early stages, both at the tertiary diabetes clinic and at the primary health care centre level.

Introduction of mandatory eye screening for all persons attending the diabetic clinic can greatly help identify potentially blinding conditions, including DR [13]. However, identification alone will not serve the purpose, unless appropriate treatment facilities and infrastructure are created at the eye clinic. The number of patients, who had severe non-proliferative retinopathy and proliferative retinopathy, requiring laser treatment, was as high as 9% in the study group. This suggests that the hospital management should explore the possibilities of establishing a medical retina unit to provide laser treatment for those in need at this level.

Our study reports the prevalence of Visual impairment, DR among the patients attending the diabetes clinic at the tertiary centre. Our study found high prevalence of type 2 Diabetes in Zanzibar, 315 (89%) among the subjects, indicating problems in lifestyle practices and this is confirmed by the high incidence of overweight (27%) and obesity (17%) among the subjects. Our reports are higher compared to previous reports from the region, regarding the prevalence of obesity among the diabetes. It was 0.2% among males in Tanzania [14] and 21% among females in urban Cameroon [15]. This is likely to increase in the future with increasing urbanization and as lifestyles shift towards reduced exercise levels, increased stress and unhealthy foods. Interestingly, a study by Martorrel et al. noted a similar situation with South African woman, where it is culturally believed that obesity reflects health and wealth [16].

More than half of the subjects (56%) reported having a history of high blood pressure (>210/120 to 140/90 mmHg) and this indicates that there is an added risk of developing other cardiovascular diseases and a risk of getting hypertensive retinopathy [17,18] which also needs to be managed as early as possible. At the time of recruitment for the study, the fasting blood sugar was high in 300 (84%) subjects and the presenting blood pressure was raised in 207 (56%) subjects. This high prevalence of suboptimal glycemic and blood pressure control is a cause for concern [19,20] and reveals a lack of diabetes care at the primary care level; this report is almost similar to the previous report from South Africa [21].

Cataract was diagnosed in 178 eyes and 59 eyes had increased IOP of greater than 21 mmHg. There is published evidence that the risk of cataract increases with an increased duration of diabetes and severity of hyperglycemia [22]. There is also a strong positive association between diabetes with primary open angle glaucoma, the most common form of glaucoma or elevated intraocular pressure in the absence of optic neuropathy [23,24].

Persons above the age of 50 years were twice at risk of developing any DR than younger persons, with an odds ratio of 2.19 with 95% Cl (1.14 -4.25). If this could have been seen and diagnosed earlier, most of these cases would have been treated. However, utilization of eye care services was poor with 61 (30%) study subjects never accessing eye care services, despite having eye problems. The barriers to accessing eye services included inadequate health education amongst diabetic patients and health care personnel. There is no diabetic screening protocol for these patients, which is vital for the early detection of DR. Instead, the eye clinic at Mnazi Mmoja Hospital depends on self-reported eye complaints, which often means that patients present late. This indicates that currently the majority of diabetic patients will progress to visual impairment, if screening and refractive services are not implemented.

Ours is the first study that assessed the prevalence of eye problems and DR among persons with diabetes in Zanzibar. Being the only tertiary and referral centre at the country, the possibility of attracting patients from all over the country was high, which was evident from the study subjects. The sample drawn for the study represented all the geographic zones in the country. Any strategic change here to improve patient care would benefit a large section of the community across the country. Moreover, this hospital is managed by the Ministry of Health, Zanzibar, with very good linkages both upwards with other tertiary centres at the Mainland, Tanzania, and downwards with all the Primary Health Care Centres, spread over the region. The primary health care units and centres can play a more active role in future by adopting an integrated approach for both diabetes and blindness prevention, through proactive screening, identification, referral and health promotion.

The main limitation of the study was that it was a hospital based study, and not a representative sample for the whole country. Although the study would help hospital policy makers to standardize the eye screening protocol for persons with diabetes attending the hospital, it will not allow us to extrapolate the information to the rest of the population.

Another limitation of this study was the DR grading done by the ophthalmologist. The method of grading was subjective, using indirect ophthalmoscope after dilation. Although the screening sensitivity of this method by an ophthalmologist showed a sensitivity of 74% in detecting DR in earlier reports [25], an objective evaluation using fundus photography would have been ideal. However, with existing resource constraints in the country, the ophthalmologist’s grading is the only possible gold standard for DR grading. Another important limitation of the study was slitlamp biomicroscopy with 78D was not done for DR grading. This would have resulted in possible underestimation of prevalence of DR. The findings from this study would help the management to expand its infrastructure for the treatment of diabetic eye diseases.

## Conclusion

We conclude that the prevalence of diabetic retinopathy and visual impairment due to other treatable causes was found to be high among the study subjects. Based on the findings, we recommend that eye screening for diabetic persons attending the MMH diabetes clinic be included as part of the regular screening protocol to detect DR and other treatable eye problems at an early stage, thus facilitating blindness prevention. The current findings are based on individuals who accessed the MMH diabetes clinic for their regular treatment. However, the number of persons with diabetes and eye problems will be much more in the community, which indicates a need to conduct further countrywide research to establish national statistics.

## Competing interests

The authors declare that they have no competing financial or nonfinancial interests.

## Authors’ contributions

FO carried out the study, analysed the results and drafted the manuscript. SS participated in concept, design, writing, editing and review of the manuscript, RPK participated in concept, design, writing, editing and review of the manuscript, GN participated in the study and GK participated in the design of the study. All authors read and approved the final manuscript.

## Author’ information

Fatma Juma Omar (FO): Master of Community Eye Health Program Student.

Sethu Sheeladevi (SS): Visiting Adjunct Lecture, School of Optometry and Vision Science, UNSW and Associate Public Health Specialist, L V Prasad Eye Institute, Hyderabad, India.

Dr. Padmaja Kumari Rani (RPK): Visiting Adjunct Lecture, School of Optometry & Vision Science, UNSW and Retina Consultant, L V Prasad Eye Institute, Hyderabad, India.

Dr. Geng Ning (GN): Consultant Ophthalmologist (Mnazi Mmoja Referral Hospital).

Dr. George Kabona (GK): Ophthalmologist (Iringa Referral Hospital).

## Pre-publication history

The pre-publication history for this paper can be accessed here:

http://www.biomedcentral.com/1471-2415/14/81/prepub
